# EBP50 inhibits pancreatic cancer cell growth and invasion by targeting the β-catenin/E-cadherin pathway

**DOI:** 10.3892/etm.2015.2684

**Published:** 2015-08-14

**Authors:** MENGYAO JI, DIKUN FAN, LEI YUAN, YUNTING ZHANG, WEIGUO DONG, XIULAN PENG

**Affiliations:** 1 Department of Gastroenterology, Renmin Hospital of Wuhan University, Wuhan, Hubei 430060, P.R. China; 2 Department of Cardiac Surgery, Center Hospital of Nayang, Henan 473009, P.R. China; 3 Department of Information Center, Renmin Hospital of Wuhan University, Wuhan, Hubei 430060, P.R. China; 4 Department of Oncology, The Fifth Hospital of Wuhan, Wuhan, Hubei 430050, P.R. China

**Keywords:** pancreatic cancer, EBP50, growth, invision, β-catenin, E-cadherin

## Abstract

Ezrin-radixin-moesin (ERM)-binding phosphoprotein 50 (EBP50) has previously been demonstrated to be associated with the malignant transformation of numerous types of human cancer. The aim of the present study was to investigate the effect of EBP50 overexpression on pancreatic cancer and the underlying mechanism. Reverse transcription-quantitative polymerase chain reaction was used to detect the expression of EBP50 in human pancreatic cancer tissue specimens. Furthermore, pBK-CMV-HA-EBP50 and the pBK-CMV-HA vectors were transfected into pancreatic cancer cells and the effect of EBP50 upregulation on the proliferation and invasion of the cells was investigated. In addition, the effect of EBP50 overexpression on β-catenin and E-cadherin expression was evaluated. The results revealed that overexpression of EBP50 suppressed cell growth and invasion in two human pancreatic cancer cell lines. Overexpression of EBP50 also suppressed β-catenin expression and increased E-cadherin expression. Thus, the present study demonstrated that EBP50 inhibits pancreatic cancer cell growth and invasion through targeting the β-catenin/E-cadherin pathway. The results suggest that EBP50 may function as a potential tumor suppressor and thus may serve as a potential therapeutic target.

## Introduction

Pancreatic cancer has an extremely poor survival rate; it ranks as the fourth most common cause of cancer-related mortality and has a five-year survival rate of ~5% (1,2). Surgery is effectively the only potential treatment for its eradication and is suitable for only 10–20% of surgical candidates. In the majority of patients, the opportunity to carry out surgery is lost for reasons such as metastatic potential and delayed diagnosis (1,3). The lack of a novel prognostic biomarker is the most important reason for the poor outcome of patients with pancreatic cancer. Therefore, the identification of efficient prognostic biomarkers is urgently required.

The pathogenesis of pancreatic cancer has not been completely elucidated. It has been found to be associated with factors including the activation of oncogenes, dysfunction of tumor suppressor genes, and aberrant activation of signal transduction pathways (4). β-catenin, which is an important effector in the Wnt signaling pathway, has an important role in cell-cell adhesion (5). Membrane-associated proteins that are formed by the binding of E-cadherin to β-catenin are involved in the regulation and provision of cellular adhesion (6). In addition, cell motility, including metastasis and invasion, is also regulated by membrane-associated adhesion proteins. In association with the extracellular matrix and actin cytoskeleton, membrane-associated adhesion proteins play crucial roles in numerous biological signal transduction pathways (7). Dysfunction of β-catenin and E-cadherin has been observed in various types of tumor, including breast cancer, colorectal cancer and pancreatic cancer (7–9).

Ezrin-radixin-moesin-binding phosphoprotein-50 (EBP50, also known as NHERF1) is a 55-kDa phosphoprotein, which is a member of the family of PDZ scaffolding proteins (10). The main site of localization of EBP50 is at the apical plasma membrane in human epithelial tissues (11). The loss of normal apical membrane expression of EBP50 and/or its distribution to the cytoplasm and nuclear overexpression have been observed in several types of human cancer (10). EBP50 has been indicated to be a tumor suppressor in several types of tumor (10). Previous studies conducted by the authors of the present study have demonstrated that the downregulation of EBP50 expression promotes the growth of gastric and pancreatic cancer cells (12,13), and that the overexpression of EBP50 regulates the apoptosis of pancreatic cancer cells by decreasing the expression levels of Bcl-2 (11).

In the present study, the aim was to explore the expression of EBP50 in pancreatic cancer tissue, the effect the overexpression of EBP50 played in pancreatic cancer cell growth and invasion and the underlying mechanism. The pBK-CMV-HA-EBP50 plasmid was used to upregulate EBP50 expression in pancreatic cancer cells and identify whether β-catenin/E-cadherin is directly targeted by its effects on the growth and invasion of pancreatic cancer cells.

## Materials and methods

### 

#### Human tissue specimens

Pancreatic cancer specimens were obtained from 120 patients of Renmin Hospital of Wuhan University (Wuhan, China) from 2003 to 2013 who were diagnosed with pancreatic cancer and had not received any preoperative chemotherapy or radiotherapy. The patients comprised 54 females and 66 males, aged from 25–82 years. A total of 60 surgical samples of pancreatic cancer and matched non-tumor tissues were collected for analysis by reverse transcription-quantitative polymerase chain reaction (RT-qPCR). World Health Organization criteria were used for the diagnosis of pancreatic cancer and the pathological tumor-node-metastasis (pTNM) staging system was applied to define the tumor stage and clinicopathological classification. Permission for the study was granted by the Ethics Committee of Wuhan University, acquired consent was obtained from every patient and the study was performed according to the principles of the Declaration of Helsinki.

#### Immunohistochemistry

Followed the manufacturer's instructions, immunohistochemical (IHC) staining of paraffinized sections was performed with anti-EBP50 polyclonal rabbit antibody (1:800; NB-300-536; Novus, Saint Charles, MO, USA). First, the pancreatic cancer tissues were fixed in 10% buffered formalin, then embedded in paraffin. After cutting into 3-µm sections and deparaffinizing in xylene, the tissues were rehydrated in several descending ethanol concentrations, incubated in 0.03% hydrogen peroxide for 10 min and then incubated in 10 mM sodium citrate buffer for 15 min for antigen retrieval. Initial blocking was followed by incubation with the anti-EBP50 antibody overnight at 4°C, three-washes with phosphate-buffered saline (PBS)-Tween-20 (T) and a second blocking step followed by incubation for 1 h at 37°C with goat anti-rabbit IgG-PerCP-Cy5.5 biotinylated secondary antibody (1:400; sc-45101; Santa Cruz Biotechnology, Inc., Santa Cruz, CA, USA). Finally, the sections were washed with PBS-T, blocked again, then incubated with diaminobenzidine (DAB) chromogen (DAB kit; Fujian Maixin Biological Technology Ltd., Fujian, China) and counterstained with hematoxylin prior to mounting. Isotype-matched irrelevant antibody was substituted for the primary antibody to act as a negative control.

#### Scoring of IHC

All stained tissue specimens were scored separately by two pathologists, who were blinded to the clinical or clinicopathological features of the specimens. The slides were scanned at low magnification (x100 objective) and confirmed under high magnification (x200 objective). The percentage of positive stained cells in 10 representative microscopic fields was evaluated as: 0, <5%; 1, 5–25%; 2, 25–50%; and 3, >50%. The intensity of staining was scored as: 0 (none), 1+ (mild), 2+ (moderate), or 3+ (intense) according to a previous study (13,14).

#### RT-qPCR

Total RNA was extracted from fresh pancreatic cancer tissues and paired adjacent non-tumorous samples using TRIzol reagent (Invitrogen Life Technologies, Carlsbad, CA, USA). According to the manufacturer's instructions, the Reverse Transcription system kit (Invitrogen Life Technologies) was used to reverse-transcribe the RNA to first-strand complementary DNA (cDNA). Corresponding levels of β-actin and EBP50 mRNA were detected by RT-qPCR using the 7500 Real-Time PCR System (Applied Biosystems Life Technologies, Foster City, CA, USA). The PCR was run for 95°C for 3 min, followed by 40 cycles of 95°C for 3 sec and 60°C for 30 sec. β-actin was used as a normalization control for EBP50 mRNA. The 2^−ΔΔCt^ method was used to quantify the relative levels of gene expression and each sample was analyzed in triplicate. The qPCR primers sequences were as follows: EBP50, forward: 5′-AGG AGT GCC TGA GTA GTC GCC AGT CAC CTG GGT CTG AGG GGC CGA CGTC-3′ and reverse: 3′-TCA GGC ACT CCT GCT TTC TTG ACC GGA CCG AAC CTG ATC A-5′; β-actin, forward: 5′-GTG ACG TTG ACA TCC G-3′ and reverse: 5′-GAG CGT TTG TTG TAC CT-3′.

#### Cell lines and transient transfection

The human pancreatic cancer cell lines PANC-1 and SW1990 were obtained from the Cell Bank of the Shanghai Institutes for Biological Sciences (Shanghai, China). Cells were maintained in HyClone™RPMI-1640 medium with 10% fetal calf serum (Gibco-BRL, Grand Island, NY, USA) and were cultured at 37°C with 5% CO_2_. The pBK-CMV-HA empty vector was obtained from Santa Cruz Biotechnology, Inc. (Dallas, TX, USA) and Dr Randy Hall from Emory University (Atlanta, GA, USA) provided the pBK-CMV-HA-EBP50 plasmid. Cells were seeded in 6-well plates at a concentration of 2×10^5^ cells/well and cultured in medium without antibiotics for 24 h prior to transfection. Lipofectamine® 2000 (Invitrogen Life Technologies) was used to conduct the transfection in accordance with the manufacturer's instructions. Cells were transiently transfected with pBK-CMV-HA-EBP50, pBK-CMV-HA or negative control (NC). After 24 h incubation at 37°C and 5% CO_2_, the cells were trypsinized and reseeded into a 12-well plate and the medium was replaced with fresh normal culture medium with G418 solution (Gibco-BRL) to select the stable transfected cell clones. The transfection efficiency of each cell clone was examined by western blot analysis (9,11).

#### Cell proliferation assay

The analysis of cell proliferation and viability was performed using a cell-counting colorimetric assay (CCK-8; Dojindo Molecular Technologies, Inc., Kumamoto, Japan). As recommended by the manufacturer, three independent experiments were conducted for each set of conditions. After washing with ice-cold PBS twice, the cells were collected by trypsinization, and then seeded on a 96-well plate at a final density of 5×10^3^ cells/well for counting. A CCK-8 kit was used to assess the cell viability at 24, 48 and 72 h. The absorbance at 450 nm was measured using a plate reader (Bio-Rad Laboratories, Inc., Hercules, CA, USA).

#### Colony formation assay

The colony formation assay was used to detect the anchorage-independent growth of the transfected cells. The cells were plated in a 6-well plate at a final density of 1×10^4^ cells/well with Dulbecco's modified Eagle's medium (DMEM) agarose medium with fetal bovine serum (FBS) and SeaPlaque™ agarose (all from Gibco-BRL). After 30 min at room temperature, the plate was hardened and then incubated at 37°C with 5% CO_2_. When the cell colonies were formed with a size >0.1 mm, phase contrast microscopy was used to calculate and photograph the result.

#### Cell invasion assay

Cell invasion assays were performed in Transwell chambers (24-well insert, pore size 8 µm; BD Biosciences, Bedford, MA, USA). The Transwell with a filter membrane having 8.0-mm pores was inserted into a 24-well plate (bottom chamber). After being suspended in serum-free medium, cells were seeded onto the top chamber in 400 µl serum-free medium. Simultaneously, 800 µl DMEM containing 10% FBS was added to the lower chambers of the Transwell plate to act as a chemoattractant. A wet cotton-tipped swab was used to remove the non-invaded cells from the upper surface. After fixation with formalin, the chambers were stained with crystal violet for 30 min. The number of cells that penetrated to the lower surface of the filter was counted and images were captured (magnification, x100) in five random fields. The data were presented as a percentage of the invaded cells in the control and each experiment was repeated three times.

#### Western blot analysis

Cells were washed with PBS then collected and lysed on ice in RIPA buffer (Thermo Fisher Scientific, Inc., Rockford, IL, USA). The BCA Protein assay kit (Pierce Biotechnology, Inc., Rockford, IL, USA) was used to measure the protein concentration. The protein fractions were denatured at 100°C by suspension in loading buffer. Total proteins were separated equally on 10% SDS-PAGE gels and electro-transferred to nitrocellulose membranes for 2 h at 4°C. Then the membranes were blocked in 5% fat-free milk in TBS-T buffer at room temperature for 2 h. Rabbit anti-EBP50 antibody (1:1,000; Abcam, Cambridge, MA, USA), rabbit anti-β-catenin (1:1,000; Abcam), mouse anti-E-cadherin (1:1,000; Abcam) and mouse anti-β-actin (1:1,000; Abcam) primary antibodies were added and the membranes were incubated overnight at 4°C. The secondary horseradish peroxidase-conjugated anti-rabbit antibodies (Sigma-Aldrich, St. Louis, MO, USA) were then incubated with the membranes for 1 h at 4°C. The bands were detected by enhanced chemiluminescence (ECL; Amersham Pharmacia Biotech, Piscataway, NJ, USA) and quantified by densitometry using UN-SCAN-IT software (Silk Scientific Corp., Orem, UT, USA). β-actin was used as an internal control.

## Results

### 

#### Expression of EBP50 is decreased in pancreatic cancer tissues

**Figure 1.**
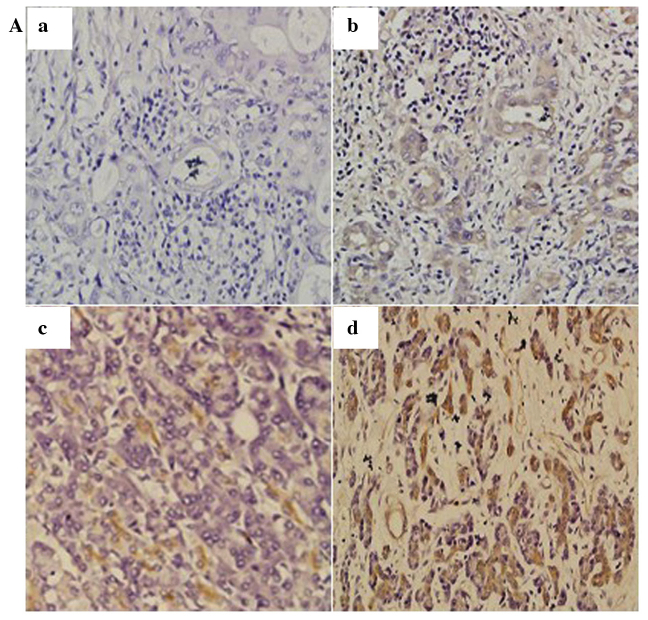

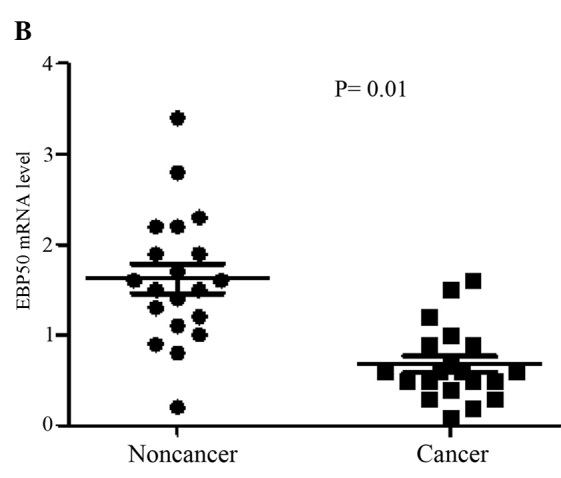
(A) Immunohistochemical analysis of EBP50 in pancreatic cancer tissues. (a) No EBP50 protein expression; (b) a positive case scoring 1+; (c) a positive case scoring 2+ and (d) a positive case scoring 3+ (original magnification, x200). (B) EBP50 mRNA levels were determined by reverse transcription-quantitative polymerase chain reaction and EBP50 expression was found to be significantly decreased in pancreatic cancer tissues compared with noncancerous tissues. EBP50, ezrin-radixin-moesin-binding phosphoprotein 50.

The expression of EBP50 was detected in pancreatic cancer tissues and corresponding non-tumor tissues. IHC results demonstrated that EBP50 was expressed in the majority of the pancreatic cancer tissues (75/120, 62.5%); 38 tissue samples (31.67%) were scored as 1+, 22 (18.33%) cases were scored as 2+ and 15 (12.5%) cases were scored as 3+ (Fig. 1A). In addition, the RT-qPCR data indicated that EBP50 mRNA expression was significantly decreased in pancreatic cancer tissues compared with the levels in corresponding normal tissues (Fig. 1B).

#### Overexpression of EBP50 represses pancreatic cancer cell growth

PANC-1 and SW1990 cells were transfected with pBK-CMV-HA-EBP50 or pBK-CMV-HA vector to establish transfected cells. G418 solution was then used to screen the stably transfected cells. To evaluate the transfection efficiency, western blotting was used to determine the protein expression of EBP50 in the pancreatic cancer cells [this data has been published previously (11)]. It was observed that EBP50 protein was upregulated in pBK-CMV-HAEBP50-transfected group compared with the levels in the pBK-CMV-HA vector-transfected cells and untreated groups.

**Figure 2.**
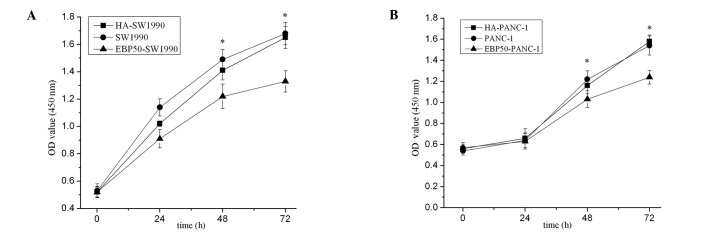
Overexpression of EBP50 suppressed cell growth in two human pancreatic cancer cell lines as determined by cell counting kit-8 (CCK-8) assay. Absorbance values were measured at 450 nm with a microplate reader. (A) SW1990 cell and (B) PANC-1 cell groups. All experiments were performed in triplicate. Data are presented as mean ± standard deviation. * P<0.05 vs. the corresponding control group. EBP50, ezrin-radixin-moesin-binding phosphoprotein 50; HA-SW1990, SW1990 cells transfected with pBK-CMV-HA empty vector; EBP50-SW1990, SW1990 cells transfected with pBK-CMV-HV-EBP50.

**Figure 3.**
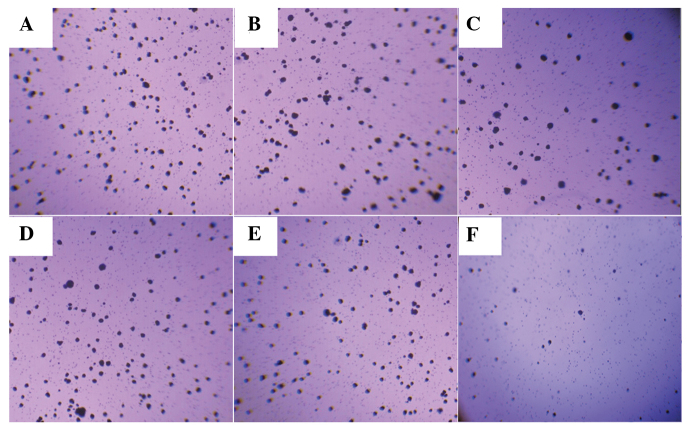
Anchorage-independent growth comparison of parental, pBK-CMV-HA-EBP50- and pBK-CMV-HA-transfected cells as evaluated by colony formation assay. (A-C) Representative data of SW1990 cells: (A) SW1990; (B) HA-SW1990 and; (C) EBP50-SW1990 cells. (D and E) Representative data of PANC-1 cells: (D) PANC-1; (F) HA- PANC-1 and; (F) EBP50- PANC-1 cells. Magnification, x100. EBP50, ezrin-radixin-moesin-binding phosphoprotein 50; HA-SW1990, SW1990 cells transfected with pBK-CMV-HA empty vector; EBP50-SW1990, SW1990 cells transfected with pBK-CMV-HV-EBP50.

CCK-8 assays and a colony formation assay were used to assess the potential effects of EBP50 overexpression on cell growth and anchorage-independent growth. The number of viable cells at different times were measured *in vitro*. The results showed that the pBK-CMV-HA-EBP50-transfected cells had markedly reduced cell growth (Fig. 2) and anchorage-independent growth (Fig. 3) compared with the untransfected cells, whereas the empty vector had no effect on pancreatic cancer cell growth.

#### Overexpression of EBP50 inhibits cancer cell migration and invasion

**Figure 4.**
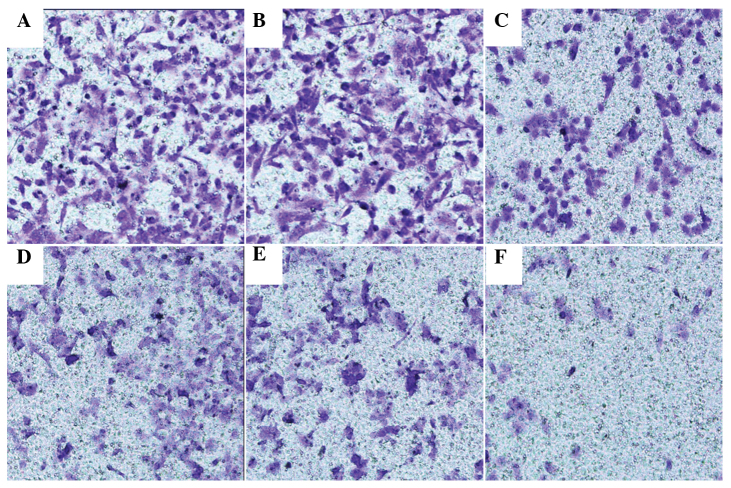
Overexpression of EBP50 inhibited the migration and invasion of pancreatic cancer cells. (A-C) Representative data of SW1990 cells: (A) SW1990; (B) HA-SW1990 and; (C) EBP50-SW1990 cells. (D and E) Representative data of PANC-1 cells: (D) PANC-1; (F) HA- PANC-1 and; (F) EBP50- PANC-1 cells. Magnification, x200. EBP50, ezrin-radixin-moesin-binding phosphoprotein 50; HA-SW1990, SW1990 cells transfected with pBK-CMV-HA empty vector; EBP50-SW1990, SW1990 cells transfected with pBK-CMV-HV-EBP50.

Transwell assay was used to explore whether the overexpression of EBP50 restrained the invasion and migration of pancreatic cancer cells. In SW1990 cells, the number of cells transfected with pBK-CMV-HA-EBP50 that migrated was less than that of the empty vector-transfected and untreated cells. In PANC-1 cells, the number of cells transfected with pBK-CMV-HA-EBP50 that migrated was less than that of the empty vector-transfected and untreated cells. No significant difference was observed between the migration and invasion of untreated and empty vector-transfected PANC-1 and SW1990 cells (Fig. 4). The data indicate that the overexpression of EBP50 significantly decreased the invasive ability of pancreatic cancer cells.

#### Expression of β-catenin and E-cadherin in pancreatic cancer cells

**Figure 5.**
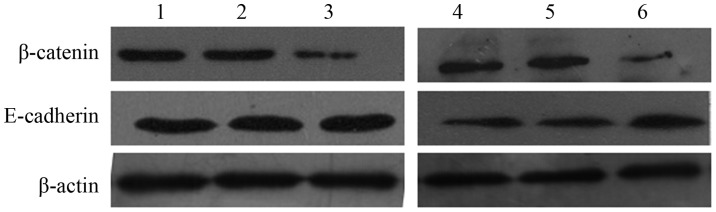
β-catenin, and E-cadherin detected by western blotting. Representative data of: Lane 1, SW1990 cells; lane 2, HA-SW1990 cells; lane 3, EBP50-SW1990 cells; lane 4, PANC-1 cells; lane 5, HA- PANC-1 cells; and lane 6, EBP50- PANC-1 cells. EBP50, ezrin-radixin-moesin-binding phosphoprotein 50; HA-SW1990, SW1990 cells transfected with pBK-CMV-HA empty vector; EBP50-SW1990, SW1990 cells transfected with pBK-CMV-HV-EBP50.

To study the potential mechanisms underlying the biological effect of EBP50 in pancreatic cancer cells, the expression of β-catenin and E-cadherin was analyzed in pBK-CMV-HA-EBP50- and pBK-CMV-HA-transfected cells and untreated cells. The data showed that protein levels of β-catenin were reduced and E-cadherin were increased in EBP50-overexpressing cells compared with those in the untreated cells (Fig. 5).

## Discussion

In the present study, it was found that the expression of EBP50 was significantly decreased in pancreatic cancer tissues compared with that in noncancerous tissues. To detect the effect of EBP50 overexpression on pancreatic cancer cell growth and invasion, the pBK-CMV-HA-EBP50 plasmid was used to increase the expression of EBP50 in PANC-1 and SW1990 cells. The CCK-8 and colony formation assays showed that the growth rate of cells overexpressing EBP50 was lower than that of the two control groups. In a Transwell assay, the number of migrated cells in the EBP50-overexpressing group was significantly lower than that in the control group. These results indicate that the overexpression of EBP50 suppressed the growth and invasion of pancreatic cancer cells. This is consistent with our previous studies (10,11,13,15).

There have been many studies in which the molecular mechanisms by which EBP50 exerts its tumor suppressor functions are reported (8–12). EBP50 links with the majority of protein receptors, including the parathyroid hormone type 1, β2-adrenergic, κ-opioid, parathyroid hormone type 1 and G protein-coupled receptors, through the first PDZ domain (8,16–19). The second PDZ domain binds with β-catenin, sodium-hydrogen exchanger 3 (NHE3) and Yes-associated protein 65 (Yap 65) (10). Both β-catenin and E-cadherin have an important role in cell-cell junctions in the Wnt signaling pathway (20). Previous studies have shown that the downregulation of EBP50 can promote pancreatic cancer cell growth through increasing β-catenin expression (13,21,22). However, there have been no further studies concerning the effect of EBP50 on the β-catenin/Wnt signaling pathway and its target protein. These data demonstrate that the overexpression of EBP50 suppresses pancreatic cancer cell growth and invasion through decreasing β-catenin and increasing E-cadherin levels. Lazar *et al* have reported the observation of disorganization of E-cadherin-mediated adherens junctions and an increase in β-catenin transcriptional activity in EBP50-deficient MEFs (23). It may be hypothesized that the balance of β-catenin/E-cadherin, which is influenced by EBP50 expression, leads to pancreatic cancer development or progression.

These data indicate that EBP50 may be a promising target for therapeutic intervention in pancreatic cancer. In addition, the tumor suppressor properties of EBP50 in pancreatic cancer may be optimized by interaction with β-catenin and E-cadherin.
